# Predict Attention Deficit Hyperactivity Disorder? Evidence -Based Medicine

**DOI:** 10.5539/gjhs.v6n2p47

**Published:** 2013-11-27

**Authors:** Abdulbari Bener, Madeeha Kamal

**Affiliations:** 1 Department of Medical Statistics and Epidemiology, Hamad Medical Corporation, Dept. of Public Health, Weill Cornell Medical College, Qatar; 2 Dept. Evidence for Population Health Unit, School of Epidemiology and Health Sciences, The University of Manchester, Manchester, UK; 3 Department of Pediatrics, Hamad Medical Corporation, Qatar; 4 Dept. of Pediatrics, Weill Cornell Medical College, Qatar

**Keywords:** epidemiology, association, ADHD, vitamin D, risk factors

## Abstract

**Background:**

Attention deficit hyperactivity disorder (ADHD) is the most common behavioral disorders in children and recent studies reported a relationship between low levels of Vitamin D and incidence of ADHD.

**Aim:**

The aim of this study was to investigate the association between vitamin D deficiency and attention deficit hyperactivity disorder (ADHD). Also, to study the impact and role of vitamin D on the development of ADH in children.

**Design:**

This is a case-control study which was conducted in children below 18 years of age from June 2011 to May 2013 at the School Health and Primary Health care Clinics, Qatar.

**Methods and subjects:**

The study was based on 1,331 cases and 1,331 controls. The data collection instrument included socio-demographic & clinical data, physician diagnosis family history, BMI, and serum 25(OH) vitamin D, calcium, albumin, billirubin, magnesium, calcium, cholesterol, urea, triglyceride and phosphorus. Descriptive and univariate statistical analysis were performed.

**Results:**

Of the total number of 3470 children surveyed, 1331 of ADHD and 1,331 of healthy children gave their consent to participate in this study. The mean age (± SD, in years) for ADHD versus control children was 10.63±3.4 vs. 10.77±3.4. Overweight (7.7% vs 9.4%) and obesity (4.6% vs 7.7%) were significantly lower in ADHD children compared to their counterparts (P=0.001). Vitamin D deficiency was considerably higher in ADHD children compared to healthy children. The mean value of vitamin D in ADHD children was much lower than the normal value and there was a significant difference found in the mean values of vitamin D between ADHD (16.6±7.8 with median 16) and control children (23.5±9.9) (p<0.0001) and with median 23 (p = 0.006). Mean values of Calcium and phosphorous were significantly higher in control compared to ADHD children (p<0.001). 1331 of all ADHD children had 19.1% had severe vitamin D deficiency (< 10 ng/ml), 44.9% has moderate insufficient levels (between 10-20 ng/ml), 27.3% has mild insufficient levels (between 20-30 ng/ml) and only 8.1% of ADHD had sufficient serum vitamin D levels (>30 ng/ml). Multivariate logistic regression analysis revealed that household income, poor relationship between parents, mothers’ occupation, consanguinity, BMI in percentiles, low duration of time under sun light, physical activity, low serum calcium level and low vitamin D level were considered as the main risk factors associated with the ADHD after adjusting for age, gender and other variables.

**Conclusion:**

The study showed that vitamin D deficiency was higher in ADHD children compared to healthy children. Supplementing infants with vitamin D might be a safe and effective strategy for reducing the risk of ADHD, but, further genomic and some other test and relevant studies need to be done.

## 1. Introduction

Attention deficit hyperactivity disorder (ADHD) is one of the most common disorders in school aged children (Barbaresi et al., 2002; St Sauver et al., 2004; Purper-Ouakil et al., 2004;Bener et al., 2008; Sayal et al., 2006a; Sayal et al., 2006b; Barkley, 2006). ADHD has very important and wide effects on the functioning and development of affected children as well as having considerable impact on others including family members, peers, and teachers (Sayal et al., 2006a; Sayal et al., 2006b; Wehmeier et al., 2010). It is one of the leading causes of academic underachievement in school as well as disruptive behaviours (Bener et al., 2008; Sayal et al., 2006a; Sayal et al., 2006b). During the last three decades, many articles have raised concerns about the issue of childhood hyperactivity and neurobehavioural disorders (Barbaresi et al., 2002; St Sauver et al., 2004; Purper-Ouakil et al., 2004; Bener et al., 2008; Sayal et al., 2006a; Sayal et al., 2006b). Attention deficit hyperactivity disorder, the most prevalent neuropsychiatric disorder worldwide in developed and developing countries, affects between 5% and 12% of school-aged children and reported some epidemiologic studies have yielded various rates of ADHD symptoms ranging from 3.2% to 17% (Bener et al., 2008; Lahat et al., 2011; Bener et al., 2006; Swanson et al., 2007; Faraone et al., 2005). ADHD is still not completely understood and remains under scientific study (Bener et al., 2006; Bener et al., 2008; Sayal et al., 2006a), meanwhile, the pathology and physiology aspect of ADHD is complex and not completely understood (Lahat et al., 2011; Bener et al., 2006; Swanson et al., 2007; Faraone et al., 2005). Unfortunately, in developed and developing countries, the majority of children who have ADHD are undiagnosed and there is limited recognition of child mental health problems in primary care. Teachers may be well placed to identify unrecognised children and to facilitate their referral to specialist medical services. Indeed, all neuropsychiatric disorders are thought to be caused by a complex and it might be a combination of genetics, environmental, and biological factors (Bener et al., 2006; Bener et al., 2008, Goyette et al., 1978; Faraone et al., 2005; Miller et al., 2004). Therefore, the proposed etiologies related to prenatal and prenatal risk factors, genetics and ADHD neurobiological deficits may all be involved in the pathphysiology of ADHD in different individuals (Bener et al., 2008, Sayal et al., 2006a; Lahat et al., 2011).

Further, children with ADHD may have functional impairment across multiple settings including home, school and social relationships (Bener et al., 2006; 2008). ADHD has also been shown to have long-term adverse effects on social-emotional development (Wehmeier et al., 2010), vocational success and academic performance (Purper-Ouakil et al., 2004; Bener et al., 2008). Furthermore, ADHD are observed and associated with an increased risk for accidents among children (Lahat et al., 2011). Prospective studies also indicate that children affected by ADHD are at a high risk of developing co-morbid disorders as well as impaired social adjustment (Swanson et al., 2007).

Vitamin D deficiency is a major health problem and has been noticed in many parts of the world (Prentice, 2008; Holick et al., 2012). It is not restricted to sunshine-limited regions of the globe and is still commonly seen in sunshine-abundant areas (Bener et al., 2009 a-b; Dawodu et al., 2002). The exact causes of such a high rates are not understood or not known. Perhaps, it might be due to vitamin D deficiency during pregnancy (Bener et al., 2013a), poor oral supplementation of vitamin D during childhood, and restricted exposure to sunshine might be risk factors for Vitamin D deficiency in children in this region (Prentice, 2008, Bener et al., 2009a-b).

The association between Vitamin D deficiency and ADHD in young children has never been reported in the literature. To the best of our knowledge, this is the first study to investigate the association between circulating levels of vitamin D and ADHD and associated risk factors.

## 2. Subjects and Methods

This is a case-control study matched by age and nationality which was designed to investigate association between vitamin D and ADHD in children below 18 years of age. The survey was conducted in the period from June 2011 to May 2013.

The International Review Board’s (IRB) approval was provided by the Research Ethics Committee, Hamad Medical Corporation, with the ethical standards laid down in the 1964 Declaration of Helsinki.

The sampling plan was stratified with proportional allocation. Stratification allowed both urban and semi-urban areas to be proportionally represented. The list of names of government schools was obtained from the Office of Director of General Education, Ministry of Education. Government schools were segregated according to gender. There were 94,985 children enrolled in government and private primary schools for boys and girls and 38,622 children enrolled in government and private intermediate or preparatory schools for boys and girls and further 34,132 children enrolled in government and private secondary schools for boys and girls in Qatar. To fulfill the objective of the current study, the study was based on 1331 cases and 1331 controls.

### 2.1 Data Collection

#### 2.1.1 Selection of ADHD Subjects

The diagnosis of ADHD was based on physician-diagnosis (American Academy of Pediatrics, Committee on Quality Improvement, and Subcommittee on attention-deficit/hyperactivity disorder). The Conners’ teacher scale was used to screen ADHD symptoms among children (Bener et al., 2006; 2008; Goyette et al., 1978; Miller et al., 2004; Purper-Ouakil et al., 2004) and teacher rating scales are also an important part of the evaluation and diagnosis. Teacher rating scales provide necessary information about the child in the school setting. We used Symbiosis National Aptitude Test (SNAP) questionnaire and or Conner’s filled by both parents and teachers in addition to the clinical history and school performance. Control subjects aged below 18 years were identified from Primary Health Care (PHC) Clinics and School Health as a part of cohort study a random sample of 1,750 ADHD children approached and 1,331 gave consent and participated in study with a response rate (76.1%). For controls, 1720 healthy subjects were approached and 1331 agreed to participate in this study (77.3%). The study excluded the subjects with following characteristics: Calcium supplements or vitamin D intake during the last 6 month before the study; history of epilepsy or anti-epileptic drugs since they affect vitamin D; any history of sun block use and the pubertal age since we know that behavioural problems and 25(OH) D2 are affected by puberty and use of sun block.

#### 2.1.2 Selection of Controls

Control subjects aged below 18 years were identified from community as a healthy and if not ever been diagnosed as ADHD or if they never if they never used any inhaler or ADHD medication. This group involved a random sample of 1,835 healthy subjects who visited the PHC Centers for any reason other than acute or chronic disease, and only 1,331 subjects included due to the either refusal of the mother or difficulty in drawing blood from very uncooperative subjects; with a response rate (72.5%).

All government schools are financed and operated by the government of Qatar. Since education in government schools is free, schools are overcrowded compared to private schools. Students in government schools are provided books and transport from school, but not meals. All government schools are segregated according to gender. Schools have daily classes for five days and the schools have two breaks for 15 minutes. Most of the schools have regular parent teachers meeting, but coordination of parents with teachers is poor in all aspects.

The Conners’ teacher scale was used to screen ADH symptoms among children. Teacher rating scales (Goyette et al., 1978) are also an important part of the evaluation and diagnosis. Teacher rating scales provide necessary information about the child in the school setting. The teacher also becomes a secondary informant who can judge the behaviour of the child in the context of his peers (Purper-Ouakil et al., 2004; Miller et al., 2004; Bener et al., 2006; 2008). The Conner’s’ Scale has been widely used in different countries, and it has well-established reliability and validity (Miller et al., 2004). The survey instruments were tested on 100 randomly selected students in different schools and validated. The internal consistency, test-retest, and interrater reliability of each scale was examined. Results showed best support for teacher completed scales, followed by ratings made by teaching assistants, and parent-report scales (Purper-Ouakil et al., 2004; Miller et al., 2004; Bener et al., 2006; 2008). Strong support for the internal consistency of the teacher-report measures was found, and it was quite similar to previously reported internal consistencies with typically developing children. Test-retest reliabilities of the teacher report measures were also quite good but tended to be lower than those reported for typically developing children. For teaching assistant ratings, test-retest reliabilities were adequate to very good and the internal consistency reliabilities for parent completed measures were adequate to excellent (Miller et al., 2004).

The Conners’ teacher scale contained 10 items for each of which the teacher is asked to indicate the degree of applicability to the child being assessed: not at all (score 0), just a little (score 1), much (score 2), very much (score 3). If the score of the child was from 0 up to 9 and from 10 up to 14, the child was classified as having mild and moderate ADHD respectively. The child who scored 15 or greater in Conners’ teacher scale is considered to have high score for ADHD; which might reflect the presence of ADHD. The literate parent of the children completed the questionnaire. Non-Qataris are expatriates who came to Qatar for employment, mostly from Middle East and Far East Asian countries. In this study, we have included non-Qatari children from Arab countries only. The relationship of the consanguineous parents was recorded. Consanguinity was defined as marriages between relatives, either first or second cousin.

### 2.2 Laboratory Investigation

#### 2.2.1 Blood Collection and Serum Measurements of Vitamin D

Trained phlebotomist collected venous blood sample, and serum separated and stored at -70°C until analysis. Serum 25-hydroxyvitamin D (25OHD), a vitamin D metabolite, was measured using a commercially available kit (DiaSorin Corporate Headquarter, Saluggia, Italy). The treated samples were then assayed using competitive binding radioimmunassay (RIA) technique. Subjects were classified into four categories: 1) *severe* vitamin D deficiency, 25(OH)D <10 ng/ml; 2) *moderate* deficiency, 25(OH)D 10-19 ng/ml; 3) *mild* deficiency, 25(OH)D 20-29 ng/ml; and normal/optimal level is between 30-80 ng/ml 18-19,24-25]. According to the recommendations of other studies (Bener et al., 2009 a-b; Dawodu et al., 2002; Holick et al., 2011), we categorized vitamin D levels as deficient if 25(OH)D is <20 ng/ml, insufficient if it is between 20-29 ng/ml and sufficient if >30 ng/ml. Other baseline biochemical parameters measured from the serum included vitamin D, calcium, magnesium, calcium, cholesterol, and phosphorus and parathyroid hormone levels Serum levels of these biochemical parameters were determined according to standard laboratory procedures. Furthermore, during the screening period, each patient provided a complete history and a comprehensive examination was performed.

Body mass index (BMI) was calculated as the weight in kilograms (with 1 kg subtracted to allow for clothing) divided by height in meters squared. BMI <85^th^ percentile was considered normal weight, 85^th^-95^th^ percentile as overweight and >95^th^ percentile as obese. Physical activity was measured as any body movement like walking, running, gardening etc for more than 30 minutes a day.

The student *t* test was used to ascertain the significance of differences between mean values of two continuous variables and confirmed by non-parametric Mann-Whitney test. A chi-square analysis was performed to test for differences in the proportion of categorical variables between two or more groups. Kruskal-Wallis one-way analysis of variance was used for comparison of several group means. Multiple Logistic regression analysis using the forward inclusion and backward deletion method was used to asses the relationship between dependent (ADHD symptoms) and independent variables and to adjust for potential confounders and orders the importance of risk factors (determinant) for the ADHD. The level p<0.05 was considered as the cut-off value for significance.

## 3. Results

Table 1 shows socio-demographic characteristics of the studied children according to ADHD and healthy children control subjects. Of the total number of 2,262 children surveyed, 1331 of ADHD and 1,331 of healthy children were contacted. The mean age (± SD, in years) for ADHD versus control children was 10.63±3.4 vs. 10.77±3.4. There were statistically significant differences between ADHD and healthy children control subjects with respect to BMI (p<0.001) and daily physical activity (p<0.001).

**Table 1:** Socio-demographic characteristics of studied ADHD and control subject (N=2662)

Variables	ADH Symptoms Children =1331 n(%)	Healthy control Children N=1331 n(%)	P value
**Age group (in years)**			
5-10 Years old	542(40.7)	538(40.4)	0.556
11-13 Years old	511(38.4)	493(37.0)	
14-18 years old	278(20.9)	300(22.5)	
**Sex**			
Male	963(72.4)	766(57.6)	<0.001*
Female	368(27.6)	565(42.4)	
**Nationality of student**		
Qatari	669(50.3)	704(52.9)	0.175
Non Qatari/Arabs	662(49.7)	627(47.1)	
**Occupation of mother**		
Housewife	487(36.6)	563(42.3)
Sedentary/Professional	278(20.9)	305(22.9)	<0.001
Clerk	282(21.2)	246(18.5)
Businesswomen	284(21.3)	217(16.3)
**Occupation of Father**			
Not working	98(7.4)	107(8.0)	0.857
Sedentary/Professional	444(33.4)	422(31.7)	
Clerk	151(11.3)	143(10.7)	
Businessman	342(25.7)	367(27.6)	
Government officer	296(22.2)	292(21.9)	
**Educational level of mother**		
Illiterate	208(15.6)	191(14.4)
Primary	227(17.1)	252(18.9)	
Intermediate	337(25.3)	380(28.5)	0.030
Secondary	309(23.2)	251(18.9)	
University and above	250(18.8)	257(19.3)	
**Educational level of father**		
Illiterate	73(5.5)	81(6.1)	0.514
Primary	182(13.7)	178(13.4)	
Intermediate	269(20.2)	255(19.2)	
Secondary	384(28.9)	420(31.6)	
University and above	423(31.8)	397(29.8)
**Income ($ US Dollars)**		
<$3,000	393(29.5)	447(33.6)	0.040
$3,000-$6,000	520(39.1)	514(38.6)	
>$6,000	418(31.4)	370(27.8)
**Daily physical activity (≥30 minutes)**	587(42.9)	702((52.7)	<0.001
**BMI Group**			
<85^th^ percentile	1168(87.8)	1104(82.9)	
85^th^ -95^th^ percentile	102(7.7)	125(9.4)	0.001
>95^th^ percentile	61(4.6)	102(7.7)	
**Consanguinity**			
Yes	492(37.0)	432(32.5)	
No	839(63.0)	899(67.5)	0.015

Table 2 presents baseline chemistry biomarker values among ADHD and control children. The study revealed that vitamin D deficiency was considerably higher in ADHD children compared to healthy children. The mean value of vitamin D in ADHD children was much lower than the normal value and there was a significant difference found in the mean values of vitamin D between ADHD (16.6±7.8 with median 16) and versus control children (23.5±9.9) (p<0.0001) and with median 23 (p=0.006).

**Table 2:** Comparison of family characteristics and school performance between cases and controls

Variables	ADH Symptoms Children =1331 n(%)	Healthy Control Children N=1331 n(%)	p value
**Daily physical activity/sport**			
**Yes**	587(42.9)	702((52.7)	<0.001
**Place of living**			
Urban	1206(90.6)	1217(91.4)	0.456
Semi Urban	125(9.4)	114(8.6)	
**Physical activities**			
Yes	711(53.4)	772(58.0)	0.019
No	620(46.6)	559(42.0)	
**Type of family**			
Nuclear	909(68.3)	978(67.3)	
Extended	288(31.6)	238(17.9)	0.011
More than one	134(10.1)	115(8.6)	
**Multiple marriages**			
Yes	196(14.7)	151(11.3)	0.010
No	1135(85.3)	1180(88.7)	
**Good relationship among parents**			
Yes	1207(90.7)	1250(93.9)	0.002
No	124(9.3)	81(6.1)	
**Parents living together**			
Yes	1192(89.6)	1228(92.3)	0.018
No	139(10.4)	103(7.7)	
**Performance at school**			
Excellent	452(34.0)	509(38.2)	
Very good	441(33.1)	411(30.9)	
Good	260(19.5)	289(21.7)	0.001
Average	178(13.4)	122(9.2)	

**Table 3:** Comparison in the behaviour of the studied children with ADHD and healthy control subjects

Variables	ADH Symptoms Children N=1331 n(%)	Healthy control Children N=1331 n(%)	p value
**Obeys rule**			
Yes	971(73.0)	1024(76.9)	0.020
No	360(27.0)	307(23.1)	
**Noisy / very active**			
Yes	216(16.2)	146(11.0)	<0.001
No	1115(83.8)	1185(89.0)	
**Cry for any reason**			
Yes	188(14.1)	1106(8.0)	<0.001
No	1143(85.9)	1223(92.0)	
**Create problems**			
Yes	187(14.0)	117(8.8)	<0.001
No	1144(86.0)	1214(91.2)	
**Nervous**			
Yes	169(12.7)	114(8.6)	<0.001
No	1162(87.3)	1217(91.4)	
**Any children like him/her**			
Yes	518(38.9)	414(31.1)	<0.001
No	813(61.1)	917(68.9)	

Table 4 reveals the distribution of serum vitamin D in Qatari children with ADHD and health children control. The mean value of vitamin D in ADHD children was much lower than the normal value and there was a significant difference found in the mean values of vitamin D between ADHD (16.6±7.8) and versus control children (23.5±9.9) (p<0.001). Besides mean values of calcium and phosphorous were statistically significant higher in control healthy children compared to ADHD children (p<0.001).

**Table 4:** Clinical biochemistry baseline value among ADHD and control subject

Variable	ADH Symptoms Children N=1331 Mean±SD	Control children N=1331 Mean ± SD	P value
**Age in years**			
Boys	10.63±3.33	10.48±3.36	0.145
Girls	10.62±3.68	11.16±3.48	0.021
**Vitamin D (ng/ml)**	16.6±7.84	23.5±9.00	<0.001
**Magnesium (mmol/L)**	0.79±0.12	0.88±0.10	<0.001
**Potassium (mmol/L)**	4.61±0.50	4.56±0.55	0.013
**Calcium (mmol/L)**	2.14±0.12	2.37±0.10	<0.001
**Phosphorous (mmol/L)**	1.48±0.30	1.56±0.26	<0.001
	**n(%)**	**n(%)**	
**Vitamin D (ng/ml) levels**			
**Severe Deficiency** 25(OH)D <10 ng/ml	262(19.7)	169(12.7)	<0.001
**Moderate deficiency** 25(OH)D 10-19 ng/ml	597(44.9)	572(43.0)	
**Mild deficiency** 25(OH)D 20-29 ng/ml	364(27.3)	400(30.1)	
**Optimal** 25(OH)D 30-80 ng/ml	108(8.1)	190(14.3)	

Table 5 shows the predictors for ADHD in children using multivariate logistic regression analysis. Household income, poor relationship between parents, mothers occupation, consanguinity, BMI in percentiles, low duration of time under sun light, physical activity, low serum calcium level and low vitamin D level were considered as the main factors associated with the ADHD after adjusting for age, gender and other variables.

**Table 5:** Multivariate logistic regression analysis as predictors for ADHD children

Independent Variables	Odds Ratio	95% Confidence Interval	P Value
Poor relationship between parents	2.98	1.65-5.33	<0.001
Consanguinity	2.83	2.41-3.35	<0.001
Low duration of time under sun light	2.72	1.86-5.15	<0.001
Physical activity	2.47	1.38-3.76	0.005
Household income	1.94	1.12 – 3.36	0.019
Occupation of mother (Housewife vs. working)	1.85	1.36-2.74	0.017
BMI in Percentiles	1.38	1.15-1.54	<0.001
Serum Calcium level	0.04	0.02-0.08	<0.001
**Vitamin D Deficiency levels:**
Severe deficiency(<10ng/ml)	2.61	1.90-3.59
Moderate deficiency (10-19ng/ml)	1.74	1.33-2.27	<0.001
Mild deficiency (20-29ng/ml)	1.52	1.14-2.01
Optimum (30-80ng/ml)	1	1

## 4. Discussion

In many developed and developing countries, the majority of children who have ADHD are undiagnosed and there is limited recognition of child mental health problems in primary care. Teachers and parents may be well placed to identify unrecognized children and to facilitate their referral to medical health care services. Vitamin D deficiency and ADHD in young children has never been previously reported in the literature. Perhaps, this is the first study investigating an association between circulating levels of vitamin D and ADHD. Besides, it showed that there is a relationship between vitamin D deficiency calcium; phosphorous; magnesium; and BMI among ADHD children. Overall, there is no study performed indicate association between Vitamin D deficiency and ADHD in young children. It has been only some about 30 years since the first reports suggesting that functions of vitamin D extended well beyond its classical role in systemic calcium homeostasis (Holick, 2012). More recently a study reported that there is convincing biological or behavioral evidence linking vitamin D deficiency to brain dysfunction (McCann & Ames, 2008).

A causal relationship between micronutrient deficiencies and suboptimal brain function would have major public health implications. Large segments of the world (including the U.S.) population, particularly the poor, are known to be undernourished for a number of micronutrients (Holick, 2012; Holick et al., 2011; McCann & Ames, 2008; McCann & Ames, 2007). A major effort to address micronutrient under nutrition as an adjunct to the various programs under way to improve dietary habits, particularly of the poor, would be well justified. A large body of research suggests that an inadequate dietary supply of any of a number of essential micronutrients such as vitamin D can adversely affect brain function (Bryan et al., 2004). Some studies also suggest positive effects of Vitamin D, multivitamin and mineral supplementation on cognitive function (Holick, 2012; Holick et al., 2011; McCann & Ames, 2007; Bener et al., 2008; TheNemoGroup, 2007; Polívka et al., 2012).

Additionally, the purpose of this study was to compare long-term school outcomes (academic achievement in reading, absenteeism, grade retention, and school dropout) for children with ADHD versus those without ADHD (Barbaresi et al., 2007). Median reading achievement scores at age 12.8 years (expressed as a national percentile) were significantly different for ADHD cases and non-ADHD controls (45 vs 73). Results were similar for both boys and girls with ADHD. Median percentage of days absent was statistically significantly higher for children with ADHD versus those without ADHD, although the difference was relatively small in absolute number of days absent. Subjects with ADHD were three times more likely to be retained a grade. Similarly, subjects with ADHD were 2.7 times more likely to drop out before high school graduation (22.9%) than non-ADHD controls (10.0%). The results of this population-based study clearly demonstrate the association between ADHD and poor long-term school outcomes. This is consistent and confirmative with the current study that school performance was higher among children without ADHD compared with ADHD children.

The correct recognition of ADHD has international public health implications (Sayal et al., 2006a; 2006b). This highlights the need to improve the ability of professionals to identify which children might benefit from further assessment. Recognition of ADHD, even if accurate, may be harmful through labeling, stigmatization, scapegoating, and effects on self-perception. It has been argued that such over-medicalisation enables both adults and children to avoid taking responsibility for a range of behaviours (Timini, 2004). Current study revealed the importance of careful psycho-educational approaches for affected children and their families. Hyperactivity carries a considerable developmental risk even when considered in dimensional terms. This suggests for improvements in identification and the consideration of behavioural management approaches in the community. However, with a longer period of follow-up and anticipated referral to medical health care services, symptomatic improvement and the cost-effectiveness of such an intervention can also be investigated (Sayal et al., 2006a).

Furthermore, several biological and epidemiological evidence strongly suggests that brain development and critical brain functions can be effected by vitamin D inadequacy related to cognitive or behavioral endpoints (Holick 2012). Although there is some limited evidence for a relationship between vitamin D inadequacy and depression, or possibly schizophrenia, studies are relatively few in number and results are mixed (Bener et al., 2012; 2013b; Timini, 2004; McGrath et al., 2010; Vinh Quoc Luong et al., 2012; Polívka et al., 2012; Umhau et al., 2013).

Over the last decade, the association of vitamin D with neuro-psychiatric diseases conditions has been the focus of interest of multiple studies including those on multiple sclerosis schizophrenia (McGrath et al., 2010), Parkinson’s disease (Vinh Quoc Luong et al., 2012), depression and suicide (Umhau et al., 2013), Alzheimer’s disease (Annweiler et al., 2013), and cognitive performance in adults (Annweiler et al., 2009). Various studies indicate that vitamin D is essential for the brain as promotes normal brain development (Eyles et al., 2009; Polívka et al., 2012; Groves et al., 2013), enhances neuroprotection and modulates matrix metalloproteinases and anti-inflammatory mechanisms (Vinh Quoc Luong et al., 2012).

Strengths of our study include large sample size, as case-control designed, age, gender and ethnicity - matched, and physician-diagnosed ADHD and mental health illnesses. There are few limitations in our study including lack of data on oral intake of vitamin D and sun exposure, and the number of different types of ADHD was relatively small.

## 5. Conclusion

In Conclusion vitamin D deficiency was greater in ADHD children compared to healthy children. Also, ADHD was also associated with a higher rate of severe Vitamin D deficiency**.** The observation of low level of vitamin D in young children could be attributed to various factors such as social customs, neglecting sunlight, dietary, and the breast feeding without any vitamin D supplement. Since vitamin D intake was found very poor in children, Vitamin D supplement to infants might be an effective strategy for reducing the risk of ADHD occurrence in children.

## What’s Known on This Subject

Studies suggest convincing biological or behavioral evidence linking vitamin D deficiency to brain dysfunction. Currently, data is lacking regarding the association between vitamin D and ADHD in human beings.

## What This Study Adds

The association between Vitamin D deficiency and ADHD in young children is never been reported in the literature. To the best of authors’ knowledge, this is the first study to investigate the association between circulating levels of vitamin D and ADHD. The present study revealed that vitamin D deficiency was greater in ADHD children compared to healthy children. Supplementing infants with vitamin D might be an effective strategy for reducing the risk of ADHD occurrence.
